# Epigenetics of the Synapse in Neurodegeneration

**DOI:** 10.1007/s11910-019-0995-y

**Published:** 2019-08-23

**Authors:** Mary Xylaki, Benedict Atzler, Tiago Fleming Outeiro

**Affiliations:** 1 Department of Experimental Neurodegeneration, Center for Biostructural Imaging of Neurodegeneration, University Medical Center Göttingen, Waldweg 33, 37073 Göttingen, Germany; 2 Max Planck Institute for Experimental Medicine, 37075 Göttingen, Germany; 3 Institute of Neuroscience, The Medical School, Newcastle University, Newcastle upon Tyne, NE2 4HH UK

**Keywords:** Synapse, Neurodegenerative diseases, DNA methylation, Histone modifications, Noncoding RNAs, Neuroepigenetics

## Abstract

**Purpose of Review:**

In the quest for understanding the pathophysiological processes underlying degeneration of nervous systems, synapses are emerging as sites of great interest as synaptic dysfunction is thought to play a role in the initiation and progression of neuronal loss. In particular, the synapse is an interesting target for the effects of epigenetic mechanisms in neurodegeneration. Here, we review the recent advances on epigenetic mechanisms driving synaptic compromise in major neurodegenerative disorders.

**Recent Findings:**

Major developments in sequencing technologies enabled the mapping of transcriptomic patterns in human postmortem brain tissues in various neurodegenerative diseases, and also in cell and animal models. These studies helped identify changes in classical neurodegeneration pathways and discover novel targets related to synaptic degeneration.

**Summary:**

Identifying epigenetic patterns indicative of synaptic defects prior to neuronal degeneration may provide the basis for future breakthroughs in the field of neurodegeneration.

## Introduction

Synapses constitute a highly specialized and vital part of neuronal cells. They are the primary sites of communication between neuronal cells, and therefore, they are involved in all aspects of neuronal physiology. Proper synaptic function is a prerequisite for normal brain function, and even minor disturbances may lead to neurological disorders. In the case of neurodegenerative disorders, these disturbances lead to progressive loss of structural and functional properties of neurons and, eventually, culminate with neuronal death in more or less specific brain regions.

The idea that synaptic dysfunction is evident before neuronal degeneration has been long established and is supported by a large body of literature in various neurodegenerative disorders such as Parkinson’s disease (PD) [1], Alzheimer’s disease (AD) [2], amyotrophic lateral sclerosis (ALS) [3], or Huntington’s disease (HD) [4]. This common denominator in neurodegenerative disorders suggests possible common molecular and/or cellular mechanism for synaptic dysfunction and pathogenesis.

**Fig. 1:**
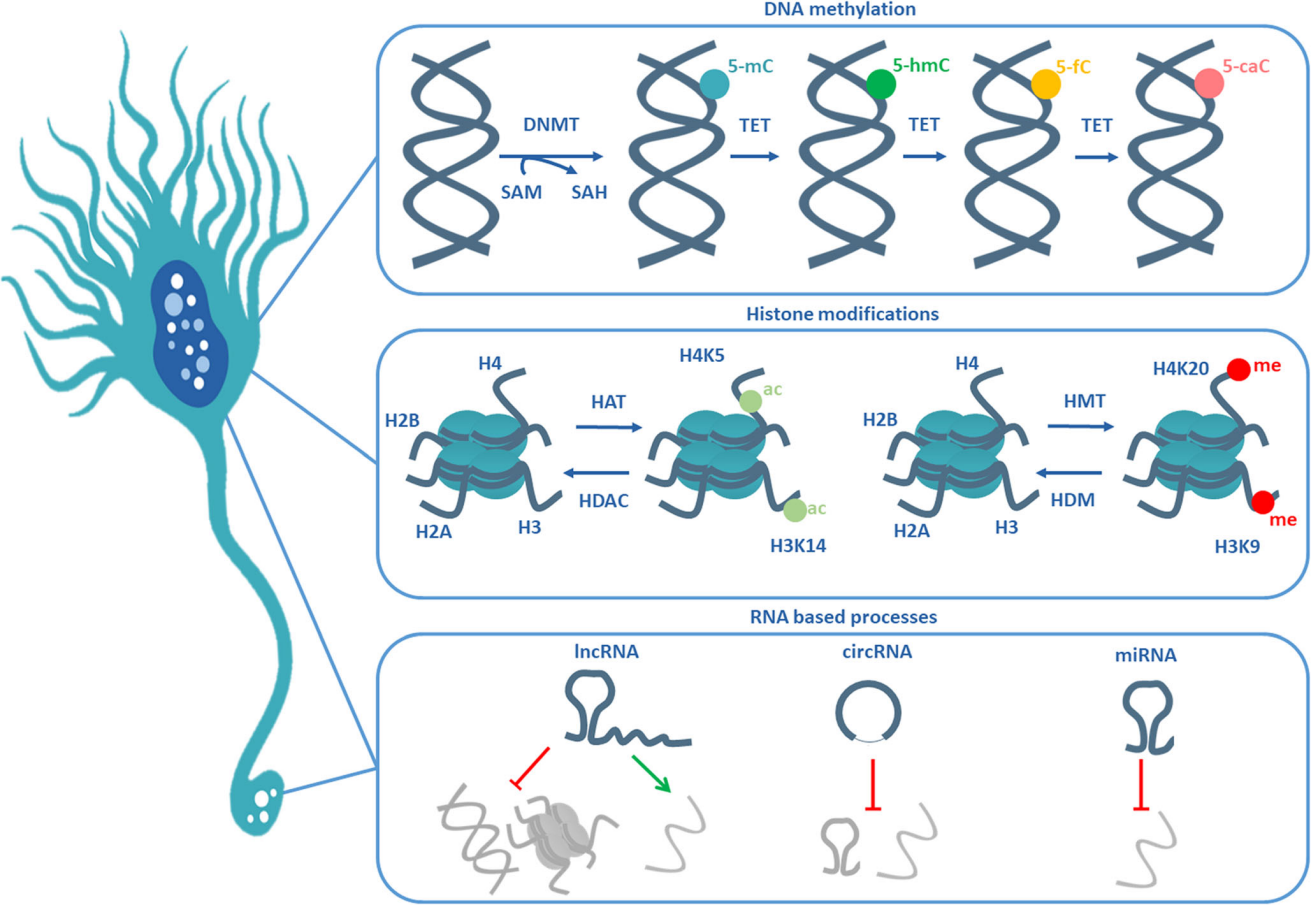
Epigenetic mechanisms. Epigenetic regulation of gene expression can be mediated by DNA methylation, by histone modifications, or by noncoding RNAs. 5-Methylcytosine (5mC) is added and controlled by DNA methyltransferase (DNMT) enzymes. This reaction is assisted by the conversion of S-adenosyl (SAM) to S-adenosyl-homocysteine (SAH). Ten-eleven translocation (TET) enzymes oxidize 5mC to 5-hydroxymethyl cytosine (5hmC), 5-formylcytosine (5fC), and 5-carboxyl cytosine (5caC), to generate cytosine again, thereby reversing the methylation code. Histone modifications are very diverse and include acetylation and methylation, which are usually installed on lysine residues (K) of one of the histones (H2, H3, H4). Histone modifications are covalent and can be reversible. Acetyl groups (ac) are added by histone acetyltransferases (HATs) and are removed by histone deacetylases (HDACs). Methyl groups (me) are added by histone methyltransferases (HMTs) and are removed by histone demethylases (HDMs). Conventionally, histone acetylation is thought to result in increased transcription, while the effects of methylation depend on the position of the modification. Long ncRNAs (lncRNAs), circular RNAs (circRNAs), and microRNAs (miRNAs) are the most studied RNA-based processes of the nervous system and exert their functions by controlling gene expression and other RNA molecules. LncRNAs can silence genes or whole chromosomes but also activate small RNAs. Circular RNAs act as molecular sponges, collecting and inhibiting mRNAs and microRNAs. MicroRNAs conventionally target complementary mRNAs inhibiting their translation or promoting their degradation

Genetics does not fully explain the vast majority of cases. Ιn PD, AD, and ALS, and, even in HD, the variability in age of onset and clinical manifestations, suggests the involvement of mechanisms that may depend on the interplay with the environment. Therefore, research has also turned to the field of epigenetics (Fig. 1), which may explain subtle changes in synaptic physiology prior to overt neurodegeneration.

Epigenetic mechanisms include any process regulating gene expression without affecting the genome sequence [5]. Conventionally, epigenetic processes comprise the chemical modifications of DNA, histones, and a wide variety of coding and noncoding RNAs, resulting in altered genomic readout and site-specific cellular phenotypes. Epigenetics is a rapidly growing field that pioneered in the study of development, aging, and cancer. In recent years, and owing also to major technological advances in next-generation sequencing, the involvement of epigenetics in neuroscience has been largely investigated. Therefore, the term “neuroepigenetics” appeared in Pubmed in 2009 and has since been widely used in the field. The contribution of epigenetic processes has been investigated in the initiation and progression of neurodegenerative disorders and has often been proposed as target for therapeutic interventions [6, 7]. Here, we review the most recent advances on our understanding of how epigenetic mechanisms may contribute to synaptic degeneration.

## DNA-Associated Epigenetic Processes in Synaptic Degeneration

DNA methylation is an abundant and widely studied type of epigenetic process. Methylated DNA is mainly inherited [8], but de novo methylation occurs during normal aging, in response to environmental factors, and in disease. Initially, DNA methylation was thought to consist of only 5-methylcytosine (5mC) [9]. This modification has a high prevalence at cytosine-phosphate-guanine (CpG) islands but can also occur in non-CpG (CpH) sites [10].

CpH methylation is present predominantly in the neuronal genome and accumulates during synaptogenesis [11]. After neuronal maturation, CpH methylation is actively maintained in the genome and is thought to restrain transcription [12]. CpH methylation is reduced in normal brain aging, but the reduction is accelerated in the prefrontal cortex of AD patients. Interestingly, AD-associated hypomethylation is mostly evident in enhancer regions of *BACE1* and genes encoding tau kinases and CDK5 neuronal activators, which enhance the production of toxic amyloid beta (Aβ) peptides and are associated with synaptic loss [13••]. Loss of CpH methylation, accompanied by a general redistribution of methylation spots, is also reported in the parietal cortex of AD patients. Importantly, methylation is increased in the ribosomal RNA (rRNA) promoter, resulting in the silencing of translation, an essential process in synapse physiology [14].

A connection between DNA methylation and the liver X receptor (LXR) transcription factor is identified in AD. In particular, activation of LXR, which is mediated by oxysterols, is downregulated in aging and neurodegenerative diseases proposed as potent therapeutic targets [15••, 16]. Pharmacological activation of LXR results in significant hypomethylation and restoration of synapse related genes, including synapsin-1, synaptophysin, and synapse- associated protein 102 (SAP-102), in the hippocampus of triple-transgenic AD (3xTg-AD) mice [17].

Manganese (Mn)-mediated epigenetic alterations in neuroblastoma SH-SY5Y cells induce differential expression of PD-associated genes. In particular, elevated Mn results in hypermethylation and downregulation of TH, PARK2, and PINK1. These genes are key players in PD and are related to early synaptic alterations [18–20].

Replication and conservation of methylation is assisted by DNA methyltransferase 1 (DNMT1), while de novo methylation is mediated by DNMT3a and DNMT3b [21, 22]. DNMTs are important modulators of gene expression in the nervous system by sustaining synaptic plasticity and function [23]. Reduced levels of DNMTs are observed in aging, AD, and PD. [24, 25] DNMTs regulate homeostatic synaptic plasticity by modulating synaptic scaling, a process that is required for fine-tuning action potential firing rates within the neuronal network. Inhibition of both DNMT1 and DNMT3a in rat cortical neurons induces up-scaling of the excitatory synaptic strength, similar to AD phenotypes associated with excitotoxicity [26–28]. Notably, DNMT1 is downregulated in frontal cortex following head-injury-mediated neurodegeneration and is linked to alterations in dendritic structural plasticity [29]. DNMTs catalyze DNA methylation by converting S-adenosyl methionine (SAM) to S-adenosyl homocysteine (SAH). SAH is thought to inhibit DNMTs via a negative feedback loop, and several dietary patterns result in SAH increases that in turn inhibit all three DNMTs [30]. Nutritionally imbalanced diets resulting in high homocysteine levels mediate the increase of SAH in mice and result in significant reductions of all DNMT enzymes, and of DNA methylation. In a mouse model of AD, such diet induces behavioral deficits that are accompanied by a reduction in the levels of synaptophysin and PSD95, and a lower number of synapses [31•].

Although 5mC is very stable, the removal of the methyl group can take place in an active or passive manner, leading to the recovery of nonmethylated cytosine. The passive manner occurs during the replication of repetitive DNA during mitosis and is known as replication-dependent dilution [32]. This does not happen in neurons, since they are postmitotic cells. The active demethylation of 5mC is mediated by the ten-eleven translocation (TET) proteins that oxidize 5mC to 5-hydroxymethyl cytosine (5hmC), 5-formylcytosine (5fC), and 5-carboxyl cytosine (5caC) [33]. 5hmC is highly abundant in the nervous system [34] and, although it does not correlate with neurogenesis and basal neuronal function, it is implicated in more complex processes that require a degree of synaptic plasticity [35]. Enhanced activity of TET and the consequent increase in 5hmC in dopaminergic neurons result in an increase in the levels of the FOXA2 transcription factor, and in enhanced survival and presynaptic function. In addition, it increases dendritic length and synapse population, as well was dopamine release [36•, 37].

## Histone-Associated Epigenetic Processes in Synapse Degeneration

DNA is packed in the nucleus in a condensed polymer that consists of histones (H1/H5, H2A, H2B, H3, and H4) and adaptor proteins, to form chromatin. Histones may undergo posttranslational modifications (PTMs) that alter their structure and biological function. The so-called histone code consists of several different types of PTMs, but 2 of them, acetylation and methylation, are covalent and dynamic [38]. Histone acetylation and deacetylation is mediated by two distinct groups of enzymes: histone acetyltransferases (HATs) and histone deacetylases (HDACs). Conventionally, histone acetylation leads to a more relaxed structure of chromatin and is associated with a higher level of gene expression. Memory consolidation in the hippocampus is tightly linked to gene expression and is often regulated by histone acetylation. The HAT Kat2a is identified in high levels in the hippocampal CA1 region that is affected during neurodegeneration in dementias. Kat2a is a regulator of synaptic plasticity and long-term memory consolidation, in a process mediated by an interaction with NF-κB signaling [39]. The Tip60 HAT is required for neuroadaptive changes induced by environmental enrichment (EE) in the *Drosophila* CNS mushroom body. Overexpression of Tip60 restores EE-induced benefits in flies overexpressing human APP by hyperacetylation of H4K45 and H4K12, leading to increased levels of synaptic proteins and upregulation of genes linked to cognitive function [40]. ANP32A is part of an inhibitory complex masking histone acetylation sites and is upregulated in human tau (htau) transgenic (Tg) mice. In this model, downregulation of ANP32A restores histone acetylation at H3K9 and H3K14, as well as at H4K5 and H4K12, and restores the levels of synaptic proteins, such as synaptophysin, synapsin-1, and glutamate receptor 1 [41]. These results indicate that imbalances in histone acetylation can lead to synaptic changes in various animal models of neurodegeneration.

Increased levels of HDAC2 are observed in AD patients and in mouse models of AD, suggesting that targeting HDAC2 and its partners may rescue neurodegeneration phenotypes [42, 43]. The transcription factor Sp3 acts as a cofactor on HDAC2 and facilitates its recruitment to synaptic genes. Sp3 is upregulated in the brains of AD patients and in mouse models of neurodegeneration. Specifically, inhibiting the formation of HDAC2-Sp3 complexes rescues synaptic gene expression alterations and function in vitro and in the CK-p25 mouse model of neurodegeneration [44].

Global hypoxia-mediated hippocampal neurodegeneration in rats induces an increase in HDAC2. Accordingly, hypoxic rats show a decrease in H3K9ac and H3K14ac accompanied by a significant decrease in SNAP-25 levels. Treatment with sodium butyrate (NaB), a broad HDAC inhibitor, rescues hypoacetylation as well as SNAP-25 levels, suggesting a regulatory function of HDAC2 in SNAP-25 expression and thus neurotransmitter release [45, 46].

The brain-derived neurotrophic factor (BDNF) is known to influence synaptogenesis, synaptic plasticity, and memory consolidation [47, 48] and is decreased in AD patients [49, 50]. Treatment with the universal HDAC inhibitor sulforaphane increases the levels of BDNF and components of the TrkB signaling cascade in mouse primary cortical neurons and 3xTg-AD mice. The subsequent increase of acetylation of H3 and H4 near the P1 promoter of the BDNF gene results in elevated levels of MAP 2 and of the synaptic proteins synaptophysin and PSD-95. These findings suggest an epigenetic effect of sulforaphane that regulates synaptic biology through BDNF signaling [51]. .BDNF is also involved in compromised dopamine signaling upon maternal deprivation (MD) in rats. MD, often linked with neurodegeneration, induces HDAC2 and reduced levels of H3K9ac. Successive elevation of the dendritic spine modulator AKAP150 lowers synaptic levels of protein kinase A and increases mBDNF. These effects can be reverted via HDAC inhibition [52].

Synaptic changes across generations can be observed in the offspring of male wild type mice which performed spatial training. Increased levels of synaptotagmin 1 (SYT1) in the offspring are accompanied by hyperacetylation of H3K9 and H3K14 at the SYT1 promoter. These changes do not occur in the offspring of 3xTg AD mice upon paternal spatial training, suggesting impairments of acetylation patterns affecting synaptic plasticity [53].

Histone methylation can lead to upregulation or downregulation of transcription, depending of the exact methylation pattern. Methylation deregulation has been observed in postmortem human samples, in animal models of neurodegenerative diseases, and in cell models expressing disease-associated proteins. H3K9me3, H3K27me3, H3K79me3, and H4K20me3 strongly bind the *C9orf72* promoter. Mutations in *C9orf72* are the most common cause of familial ALS and frontotemporal dementia (FTD) and are linked with impairments in synapse formation, morphology, and function [54•]. Upon binding to the methylated histones, transcription is reduced and the levels of *C9orf72* mRNA levels are decreased in frontal cortices and cerebella of FTD/ALS patients. Treatment with 5-aza-2-deoxycytidine, a DNA and histone methylation inhibitor, restores *C9orf72* expression in FTD/ALS fibroblasts [55].

H3K9me2 is significantly increased in the prefrontal cortex (PFC) of AD patients and in the mouse model expressing five familial AD (5xFAD) mutations. Aged 5xFAD mice display glutamate receptor alterations and impairments in synaptic transmission and memory formation, in a process regulated by an increase in the levels of euchromatic histone methyltransferases EHMT1 and EHMT2 [56•]. In contrast to findings in the PFC, H3K9me2 is decreased in the hippocampal CA1 region of AD patients, and ribosomal gene expression is altered in the CA1 and DG regions. The abundance of translation initiation factors is altered prior to neuronal cell death, therefore supporting an early defect in protein synthesis that affects dendritic branches and synapse number [57].

Overexpression of α-synuclein, a key player in Parkinson’s disease (PD) and other synucleinopathies, is associated with increased levels of H3K9 methylation in *Droshophila* and in neuroblastoma SH-SY5Y cells. Transcriptional repression through H3K9me2 results in a decrease of *L1CAM* and *SNAP25* mRNA levels, alterations that might affect synaptic function via deficits in SNARE complex assembly and neurite outgrowth [58].

The importance of a proper methylation “code” is also evident in the context of neural stem cells (NSCs). While NSCs hold a promising potential to differentiate into dopaminergic cells, which might then be used as a replacement therapy for degenerating cells in PD patients, in vitro experiments showed that the neurogenic potential of NSCs declines over time [59]. After treatment with vitamin C (VC), several histones in NSCs are hypomethylated (e.g., H3K9me3, H3K27m3) at marker gene promoter regions (e.g., *Foxa2* and *Lmx1a*), and maintain their neurogenic potential. Additionally, dopaminergic neurons differentiated from VC-treated NCSs show higher levels of synapsin and increased presynaptic dopamine release, suggesting that treatment with VC not only enhances cell survival but also presynaptic function [60•].

## RNA-Associated Epigenetic Processes in Synapse Degeneration

The multitude of forms and functions of RNA are highly attractive and intriguing. From macromolecular machines to small modulators of gene expression, RNAs are extremely important molecules in biology. Strikingly, 98% of transcribed RNAs are noncoding RNAs (ncRNAs) and are considered to participate in genomic programming [60]. The list of ncRNAs has grown tremendously and is now quite long and diverse, with new functions being assigned to the different classes [61, 62]. Among them, long ncRNAs (lncRNAs), circular RNAs (circRNAs), and microRNAs (miRNAs) are highly enriched in the nervous system [63–65] and have been associated with different aspects of neuronal development, synaptic plasticity, and function, and also with disease [66–68].

Among all epigenetic mechanisms, RNA-based processes are the only ones operating locally at synapses. The first layer of regulation of such processes lays on mechanisms such as RNA transport, local storage, and translation, all of which are dependent on RNA-binding proteins (RBPs) [69].

## Long Noncoding RNAs

LncRNAs are important players in fine tuning brain physiology as they are present in the distal dendritic spines implicated in local active duty [64, 70]. LncRNAs are greater than 200 nt in size and may be divided in two subclasses: the large intergenic noncoding RNAs (lincRNAs), transcribed from DNA sequences between genes in a manner similar to mRNAs, and the lncRNAs, transcribed from regions within genes [71, 72]. The great potential of lncRNAs is illustrated by their function: lncRNAs can silence small transcripts or entire chromosomes [73] and are proposed to have enhancer function [74]. Recently, a lncRNA, GM12371, identified in mouse hippocampal neurons, was shown to enhance the expression of synapse-related genes and control synaptic morphology and structure [75•].

The identification of such transcriptional regulators of synaptic function may constitute important markers for neurodegenerative disorders. LincRNAs HAO2-AS, EBF3-AS, AD-linc1, and AD-linc2 are upregulated in the hippocampus and entorhinal cortex of AD patients. These transcripts are particularly enriched in the nucleoplasm and are proposed to control histone-associated processes resulting in the downregulation of genes linked to signal transduction and synaptic plasticity, without affecting the levels of genes encoding scaffolding proteins [76]. Another nucleolar lncRNA, LoNA, was found to control translation by suppressing rRNA transcription and methylation, thereby leading to detrimental effects in the synapse. Downregulation of LoNA was found to strengthen memory in wild-type mice and to restore learning and memory deficits in APP/PS1 transgenic animals along with an increase in synaptic markers synaptophysin, PSD95, and glutamate receptors [77••].

Several lncRNAs are found deregulated in the substantia nigra of presymptomatic transgenic mice expressing double mutated (A30P and A53T) alpha-synuclein. Notably, these deregulated lncRNAs are associated with deficits in synaptic transmission prior to neuronal cell death [78••].

A surprising function of lncRNAs was identified in *Drosophila melanogaster*. In these flies, the lncRNA hrsω was found to physically interact with and retain the RBP FUS in the nucleus. Deletion of hrsω results in translocation of FUS to the cytoplasm and is associated with abnormal architecture of the presynapse of motor neurons [79]. These findings suggest novel molecular mechanisms associated with ALS and other neurodegenerative disorders driven by RBPs [80].

## Circular RNAs

CircRNAs belong to the short class of ncRNAs, as they are shorter than 200 nt. CircRNAs are produced in a manner similar to mRNAs, but form a 5′ end to 3′ end covalent bond to form a circle, via an anterograde splicing event [81]. CircRNAs are enriched at synapses and are dynamically altered upon synaptic plasticity [65, 82]. Our understanding of the functions of circRNAs is expanding but, thus far, they are mainly considered transcriptional regulators acting as sponges sequestering mRNAs and miRNAs besides RBPs [81].

The circRNA for HDAC9 was found to act as a sponge for mir138, which induces synaptic and cognitive impairments in mice. Notably, circHDAC9 is decreased and mir138 upregulated in the APP/PS1 mice [83••].

Interestingly, a cKO mouse model of TAR-DNA binding protein 43 (TDP-43) exhibits deregulation of circRNA processing that is accompanied by deregulation of synaptic proteins associated with plasticity, calcium signaling, and with cognitive function [84••].

To identify circRNAs involved in neurodegeneration, a mouse model of oxidative stress lacking nuclear factor erythroid 2-related factor 2 (Nrf2) was used. Correlating circRNAs, miRNAs and mRNAs revealed several coregulated partners associated with prion diseases, ALS, AD, and synaptic vesicle cycle regulation [85].

## Micro-RNAs

MiRNAs belong to the class of short noncoding RNAs being 20–23 nucleotides long. They exert their function by complementary binding to mRNA, leading to translation repression or degradation of the target mRNA [86]. Accumulating evidence underscores the importance of miRNAs in synapse development, function and plasticity [87, 88]. MiRNAs are identified both in pre- and post- synaptic compartment and have been extensively correlated with neurodegeneration [89, 90]. Synaptosomes isolated from prion protein infected mice prior to the onset of symptoms revealed an upregulation of miR-136-5p, miR-361-5p, miR-212-3p, miR-129-2-3p, miR-345-5p, and miR-124-3p, facilitating dendrite loss and gliosis [91]. The transcription repressors Bmi1, Sox11, and Zfp90 were found to be downregulated by miR-27b, suggesting that this miRNA is a major regulator of the presynaptic transcriptome in mouse cortical neurons.

Upregulation of miR-27b is also correlated with enhanced neurotransmission [92]. There are many clusters of miRNAs that act synergistically towards the establishment of certain phenotypes like the miR-132, miR-134, and miR-138 that regulate neuronal dendrites [93]. Similarly, miR-132 and miR-212 have common regulatory elements and are involved in synaptic transmission and memory formation [94]. The aforementioned clusters of miRNAs show specificity in regulating synaptic structure and function. Therefore, a better understanding of the modes of action of these and other miRNAs may provide novel insight into the molecular basis of various neurodegenerative diseases.

TDP-43 is genetically linked with familial forms of ALS and FTD and is directly implicated in RNA processing. Upon translocation to the cytoplasm, TDP-43 is involved in miRNA processing, and promotes changes in dendritic length and spine morphology that take place prior to neuronal death [95, 96]. Deep sequencing of post mortem spinal cord tissue from ALS patients revealed a downregulation of miRNAs linked to neuronal processes, such as miR-485, linked with synaptic maintenance, and miR-124 and miR-127, both of which are implicated in neural outgrowth [97].

## Conclusions

Here, we have provided a general overview of recent research on epigenetic-based processes affecting synaptic biology in the context of different neurodegenerative disorders. In short, epigenetic processes can modulate the expression of important components of synaptic function and also of genes directly associated with neurodegeneration.

Epigenetic processes have been now studied for a few years in the context of neurodegenerative diseases and are now starting to be used as targets for therapeutic intervention [98]. However, this field is still young, and requires additional research [99, 100]. Early epigenetic research focused on discovering modifications in genes identified by genome-wide association studies (GWAS) for the different neurodegenerative diseases [101]. This was initially achieved with the use of DNA microarrays [102] and has more recently progressed to the use of next generation sequencing technologies [103]. Despite tremendous recent progress, it is still unclear how a whole-cell epigenetic effect is directing a specific synaptic phenotype. This question remains still partially unanswered as the interface between epigenetics and synaptic functions is currently investigated. Ongoing and future studies will undoubtedly shed light into these processes, and may open novel avenues for therapeutic intervention and even diagnostics in neurodegenerative diseases.

## Abbreviations

3xTg-AD: Triple-transgenic AD
5xFAD: Five familial AD
5caC: 5-Carboxyl cytosine
5fC: 5-Formylcytosine
5hmC: 5-Hydroxymethyl cytosine
5mC: 5-Methylcytosine
Aβ: Amyloid beta
AD: Alzheimer’s disease
ALS: Amyotrophic lateral sclerosis
BDNF: Brain-derived neurotrophic factor
circRNAs: Circular RNAs
cDKO: Conditional double knockout
CpG: Cytosine-phosphate-guanine
CpH: Non-CpG
DNMT: DNA methylatransferase
EE: Environmental enrichment
EHMT: Euchromatic histone methyltransferases
FTD: Frontotemporal dementia
GWAS: Genome-wide association studies
H: Histone
HATs: Histone acetyltransferases
HD: Huntington’s disease
HDACs: Histone deacetylases
HDM: Histone demethylase
HMT: Histone methyl transferase
htau: Human tau
lincRNAs: Large intergenic noncoding RNAs
lncRNAs: Long ncRNAs
LXR: Liver X receptor
MD: Maternal deprivation
miRNAs: MicroRNAs
Mn: Manganese
NaB: Sodium butyrate
ncRNAs: Noncoding RNAs
Nrf2: Nuclear factor erythroid 2-related factor 2
NSCs: Neural stem cells
PFC: Prefrontal cortex
PD: Parkinson’s disease
PTMs: Posttranslational modifications
RBPs: RNA-binding proteins
rRNA: Ribosomal RNA
SAH: S-Adenosyl homocysteine
SAM: S-Adenosyl methionine
SAP-102: Synapse-associated protein 102
SYT1: Synaptotagmin 1
TDP-43: TAR-DNA binding protein 43
TET: Ten-eleven translocation
Tg: Transgenic
VC: Vitamin C
